# Artificial Intelligence-Assisted Lung Ultrasound for Pneumothorax: Diagnostic Accuracy Compared with CT in Emergency and Critical Care

**DOI:** 10.3390/tomography11110121

**Published:** 2025-10-30

**Authors:** İsmail Dal, Kemal Akyol

**Affiliations:** 1 Department of Thoracic Surgery, Faculty of Medicine, Kastamonu University, Kastamonu 37150, Turkey; 2 Department of Computer Engineering, Faculty of Engineering and Architecture, Kastamonu University, Kastamonu 37150, Turkey; kakyol@kastamonu.edu.tr

**Keywords:** pneumothorax, diagnostic imaging, ultrasonography, point-of-care systems, emergency service, artificial intelligence

## Abstract

**Simple Summary:**

Pneumothorax is a life-threatening condition that requires rapid and accurate diagnosis, especially in emergency and critical care settings. Although lung ultrasound (LUS) offers a fast and radiation-free diagnostic option, its accuracy can vary depending on the operator’s experience. This study evaluated the potential of artificial intelligence (AI) to assist clinicians by automatically detecting pneumothorax on LUS images and videos. Using transformer-based deep learning models, we compared the diagnostic performance of Vision Transformer (ViT), DINOv2, and Video Vision Transformer (ViViT) architectures. When tested on data from different patients, the DINOv2 model achieved 90% accuracy, demonstrating reliable generalization without overfitting. Furthermore, when video sequences were analyzed, both Random Forest and eXtreme Gradient Boosting classifiers trained on ViViT-derived features achieved 90% accuracy, showing that AI can effectively interpret dynamic pleural motion. These results indicate that transformer-based AI can enhance pneumothorax diagnosis by improving consistency and reducing operator dependence, supporting broader use of lung ultrasound in emergency and point-of-care environments.

**Abstract:**

**Background:** Pneumothorax (PTX) requires rapid recognition in emergency and critical care. Lung ultrasound (LUS) offers a fast, radiation-free alternative to computed tomography (CT), but its accuracy is limited by operator dependence. Artificial intelligence (AI) may standardize interpretation and improve performance. **Methods:** This retrospective single-center study included 46 patients (23 with CT-confirmed PTX and 23 controls). Sixty B-mode and M-mode frames per patient were extracted using a Clarius C3 HD3 wireless device, yielding 2760 images. CT served as the diagnostic reference. Experimental studies were conducted within the framework of three scenarios. Transformer-based models, Vision Transformer (ViT) and DINOv2, were trained and tested under two scenarios: random frame split and patient-level split. Also, Random Forest (RF) and eXtreme Gradient Boosting (XGBoost) classifiers were trained on the feature maps extracted by using Video Vision Transformer (ViViT) for ultrasound video sequences in Scenario 3. Model performance was evaluated using accuracy, sensitivity, specificity, F1-score, and area under the ROC curve (AUC). **Results:** Both transformers achieved high diagnostic accuracy, with B-mode images outperforming M-mode inputs in the first two scenarios. In Scenario 1, ViT reached 99.1% accuracy, while DINOv2 achieved 97.3%. In Scenario 2, which avoided data leakage, DINOv2 performed best in the B-mode region (90% accuracy, 80% sensitivity, 100% specificity, F1-score 88.9%). ROC analysis confirmed strong discriminative ability, with AUC values of 0.973 for DINOv2 and 0.964 for ViT on B-mode images. Also, both RF and XGBoost classifiers trained on the ViViT feature maps reached 90% accuracy on the video sequences. **Conclusions:** AI-assisted LUS substantially improves PTX detection, with transformers—particularly DINOv2—achieving near-expert accuracy. Larger multicenter datasets are required for validation and clinical integration.

## 1. Introduction

### 1.1. Clinical Background

Pneumothorax (PTX) is a life-threatening condition characterized by the accumulation of air in the pleural space, causing partial or complete lung collapse. Rapid and accurate diagnosis of PTX is critical to prevent respiratory or circulatory collapse [1]. Traditionally, chest X-ray (CXR) has been the first-line imaging modality for suspected PTX, while computed tomography (CT) serves as the diagnostic gold standard [2]. However, CXR has relatively low sensitivity for PTX, especially in occult cases or when patients are supine, often missing a significant proportion of pneumothoraces [3]. CT scans can detect even small PTXs, but they are time-consuming, costly, and expose patients to ionizing radiation [4]. In contrast, lung ultrasound (LUS) has emerged as a rapid, repeatable, and radiation-free alternative for PTX diagnosis, particularly in emergency and critical care settings [5]. A large body of evidence indicates that LUS is more sensitive than chest radiography for detecting pneumothorax, while maintaining high specificity [6]. Meta-analyses report that bedside ultrasound can achieve sensitivity as high as 86–90% and specificity 98–99% for PTX, outperforming standard chest X-rays in accuracy [7]. Accordingly, point-of-care ultrasound has become a key component of trauma protocols (e.g., the Extended Focused Assessment with Sonography for Trauma, E-FAST) and critical care algorithms for prompt PTX identification and management [8].

Despite its proven diagnostic performance, lung ultrasound’s effectiveness heavily depends on operator skill and experience. Proper image acquisition and interpretation require training in recognizing sonographic signs of PTX, such as the absence of lung sliding (often seen as the “barcode sign” on M-mode) and the absence of B-lines or lung pulse [9]. Expert sonographers can identify pneumothorax with a high degree of confidence, but ultrasound is highly operator-dependent, and its accuracy may drop in the hands of less experienced clinicians. This operator variability has traditionally impeded the widespread adoption of LUS for PTX in some settings, particularly prehospital and resource-limited environments where skilled ultrasonographers are not readily available. Surveys of critical care providers have highlighted practical barriers to mastering lung ultrasound, including steep learning curves and subjective interpretation differences [10]. There is a clear need for solutions that can support less-experienced users and standardize pneumothorax detection on ultrasound, thereby extending the benefits of LUS to a broader range of clinical scenarios (e.g., ambulance services, rural clinics, and battlefield triage).

Recent advances in artificial intelligence (AI) offer a promising avenue to address these challenges. In particular, deep learning—a family of machine learning techniques capable of automatic feature extraction from images—has revolutionized medical image analysis [11]. Convolutional neural networks (CNN) and related models can learn to recognize complex patterns given sufficient training data, and have already shown remarkable success in interpreting chest radiographs and CT scans [12,13]. Applying AI to lung ultrasound is a logical next step to mitigate operator dependence: an AI-driven system could automatically identify sonographic signs of PTX (such as the loss of pleural motion) and alert providers in real time [14]. In theory, this could enable even novice ultrasound operators to achieve diagnostic accuracy approaching that of seasoned experts. However, developing robust AI for LUS interpretation poses unique difficulties. Ultrasound images are more operator-variably and artifact-laden than X-ray/CT images, and large public datasets for lung ultrasound have historically been lacking [15]. The limited availability of labelled ultrasound data and the complexity of dynamic ultrasound artifacts (e.g., movement of pleura) have been significant barriers to training conventional neural networks for this task [16]. Nevertheless, in the past few years there has been a growing research effort exploring AI solutions specifically for pneumothorax detection using ultrasound, with encouraging results across a spectrum of experimental and clinical studies [17,18].

In this context, the present study builds upon this growing body of work to address the need for accurate and standardized pneumothorax detection in diverse clinical environments. We developed and evaluated three transformer based deep learning architectures for the analysis of point-of-care lung ultrasound data acquired in emergency and intensive care settings. By leveraging these state-of-the-art AI techniques, our goal is to enhance diagnostic accuracy, reduce operator dependency, and facilitate wider adoption of lung ultrasound in real-world clinical practice.

### 1.2. State of the Art in AI-Assisted Pneumothorax Diagnosis with Ultrasound

#### 1.2.1. Early Feasibility Studies (Animal and Phantom Models)

In the literature, early proof-of-concept studies demonstrated the feasibility of automated pneumothorax recognition on ultrasound images. Summers et al. (2016) developed a rule-based computerized diagnostic assistant that analysed B-mode clips for pleural sliding and M-mode images for the “seashore sign” [19]. In a pilot evaluation on archived trauma scans, their algorithm achieved 79% sensitivity and 87% specificity for PTX when compared to an expert panel, and notably 100% sensitivity on a subset of high-quality. This early work proved that key sonographic features of pneumothorax could be detected algorithmically. Building on that foundation, Summers et al. (2017) introduced the “iFAST” (intelligent FAST) ultrasound algorithm using a controlled porcine model of pneumothorax [20]. In that study, ultrasound videos were collected from ventilated pigs with incrementally introduced PTXs [20]. The iFAST system demonstrated extremely high performance, detecting pneumothorax with 98% sensitivity and 95% specificity on M-mode ultrasound images in the animal model. Even on raw B-mode video clips (which are more challenging due to respiratory motion and noise), the algorithm reached about 86% sensitivity and 85% specificity. These results were among the first to show that a computer algorithm could rival human-level interpretation of ultrasound for pneumothorax in a controlled setting. Subsequent experiments applied modern deep learning to the same dataset: Lindsey et al. (2019) trained deep CNN models (e.g., VGG16, VGG19, ResNet50) on the pig ultrasound data and reported near-perfect classification of pneumothorax images [21]. Their best model improved B-mode video analysis to 97–99% sensitivity/specificity, substantially higher than the original iFAST outcomes. However, it was noted that some of this impressive performance might be due to overlap between training and test data (frames from the same animal appearing in both sets), which could inflate accuracy estimates. In parallel, other groups explored complementary AI approaches. Kulhare et al. (2018) designed a deep learning pipeline to detect various lung pathologies on ultrasound; for pneumothorax, they used an InceptionV3 CNN to classify simulated M-mode images and achieved about 93% sensitivity and specificity on a test set of 35 ultrasound videos [22]. Mehanian et al. (2019) developed a suite of AI methods (including M-mode analysis, feature fusion, and Long-Short Term Memory for temporal modelling) to recognize absent lung sliding, using ultrasound data from four pigs [23]. Their best-performing classifier had a mean sensitivity of 78–84% and specificity 82–87% for pneumothorax detection in a rigorous four-fold cross-validation (leaving one animal out for each fold). These studies collectively established a proof of concept that AI algorithms can automatically identify the ultrasonographic signatures of pneumothorax in both still images and dynamic clips.

#### 1.2.2. Deep Learning on Simulated and Augmented Data

A consistent challenge in developing deep learning models for LUS has been the scarcity of large, annotated datasets. To address this challenge, researchers have turned to creative solutions such as simulation and data augmentation. Boice et al. (2022) pioneered the use of synthetic ultrasound phantoms to generate training data for pneumothorax AI [4]. They constructed a realistic tissue phantom with a pleural–air interface and simulated rib cage, which produces ultrasound images mimicking real lung scans with and without PTX. Using this phantom, they trained a deep classification network and then tested it on actual pig ultrasound images. Notably, a model trained solely on synthetic data successfully identified pneumothorax in swine scans acquired in vivo, achieving an accuracy of about 82%. Furthermore, by enriching the training set with augmented images (varying brightness, contrast, etc.), they improved the test accuracy to 93.6%, nearly on par with models trained on real data. This study highlighted the value of affordable, reproducible ultrasound phantoms for generating large volumes of labeled data that deep learning requires, thereby circumventing the need for labor-intensive animal experiments or clinical image collection. The use of phantoms and synthetic data has opened the door for more robust and generalizable AI models in ultrasound by providing an unlimited supply of training examples, while also underscoring the importance of careful validation on real-world scans.

#### 1.2.3. Clinical Data-Based AI Models

As the field matures, recent investigations have shifted focus toward clinical ultrasound data and real-time implementation. Jascur et al. (2021) published one of the first deep learning studies on human lung ultrasound videos for pneumothorax [11]. They developed a model to automatically recognize the absence of lung sliding—a critical sign of PTX—in B-mode ultrasound clips [11]. Their algorithm, based on a ResNet-18 CNN analyzing temporal sequences, detected absent sliding with a balanced accuracy of 89% (82% sensitivity, 92% specificity) in post-thoracic surgery patients. The authors demonstrated that AI was capable of interpreting pleural movement in real patient scans, rather than being limited to static images or animal data. Another notable study by VanBerlo et al. (2022) took a large-scale data approach: they aggregated lung ultrasound exams from two academic centers and trained a deep model to assess the presence of the lung-sliding artifact [17]. By converting B-mode videos into M-mode-like slices and feeding these into a custom CNN, their system achieved 93.5% sensitivity and 87.3% specificity for detecting lung sliding (corresponding to PTX detection when sliding is absent), with an area under the receiver operating characteristic (ROC) curve (AUC) of 0.973. This high accuracy was maintained on an external validation set, indicating good generalizability. Importantly, VanBerlo et al. also emphasized model interpretability, as their algorithm provided visual overlays to show where pleural motion was detected or not. Around the same time, Kim et al. (2022) described a stepwise AI-assisted ultrasound framework for PTX diagnosis, explicitly mirroring the workflow of experienced clinicians [8]. In their approach, one neural network first identifies and zooms in on the pleural line in the ultrasound image (quality assurance and pleural localization), and a second network then classifies lung sliding vs. no-sliding using M-mode data. They implemented this pipeline on a lightweight EfficientNet model to allow real-time inference on portable devices. The results were promising: both the pleural line detection and lung sliding classification achieved AUCs > 95%, and the integrated end-to-end system attained an overall AUC of 0.89 for diagnosing pneumothorax in a retrospective test set. This stepwise strategy provides an intelligible decision process (finding pleura → checking sliding) that may be easier for clinicians to trust and follow. Most recently, Qiang et al. (2025) introduced a video comprehension model for lung ultrasound, leveraging a Temporal Shift Module (TSM) network to fuse spatial and temporal features across entire ultrasound clips [18]. In what they described as a “multi-feature fusion” approach, their deep learning model considers not only lung sliding but also other sonographic indicators (e.g., comet-tail artifact changes) to emulate how a human expert diagnoses PTX from video. Tested on a sizable dataset of 164 lung ultrasound videos, their method achieved an average accuracy of 96.9%, with 99.2% sensitivity and 89.2% specificity for pneumothorax identification. This represents one of the highest-performing AI systems for PTX to date, and notably it approaches the ideal of analysing ultrasound videos in their entirety (as opposed to single frames or single sweeps), much like a clinician observing motion in real-time.

#### 1.2.4. Prospective and Comparative Validation Studies

Beyond algorithm development, investigators have begun reporting prospective and comparative studies to evaluate how AI performs against human clinicians. Clausdorff Fiedler et al. (2024) conducted a prospective diagnostic accuracy study of an AI system for real-time lung sliding detection during trauma ultrasounds [24]. In a cohort of emergency patients, the AI model was able to detect the absence of lung sliding with high sensitivity (on the order of 90–100%) but only moderate specificity, due to some false positives in cases with poor image quality or equivocal sliding. This mirrors the classic trade-off in pneumothorax diagnosis: maximizing sensitivity (to avoid missed PTXs) can come at the expense of more false alarms, an issue that AI will need to balance with clinical input. Another recent study by Yang et al. (2024) compared an AI-assisted LUS diagnostic protocol to the standard radiology work-up for pneumothorax in a hospital setting [25]. The AI-assisted lung ultrasound showed 79.4% sensitivity and 85.4% specificity for pneumothorax, which was on par with the accuracy of the attending radiologists’ assessments using CXR and CT. This suggests that an intelligent ultrasound system could potentially serve as an effective triage or screening tool, flagging pneumothoraces at the bedside with performance approaching that of formal imaging studies. It is worth noting, however, that certain clinical scenarios remain challenging—for instance, differentiating true lung sliding absence due to PTX vs. due to extensive adhesions or mainstem intubation. Advanced algorithms are being trained to recognize these nuances (such as identifying the lung point or lung pulse sign to confirm a pneumothorax) to reduce false positives [11]. Overall, the trend in current research is moving from controlled experiments to real-world validation, ensuring that AI tools maintain high accuracy in diverse patient populations and imaging conditions.

In addition to pneumothorax detection, AI is making inroads in analyzing other lung ultrasound findings, further underscoring its versatility. For example, a recent study evaluated an AI tool for automatically detecting and counting A-lines and B-lines on lung ultrasound—artifacts that are key to assessing pulmonary edema, fibrosis, or COVID-19 pneumonia severity [15]. ExoLungAI demonstrated 91% sensitivity and 81% specificity in identifying normal A-lines, and 84% sensitivity and 86% specificity in detecting pathologic B-lines, showing strong concordance with expert counts of B-line numbers. This automated quantification of B-lines provides rapid, objective monitoring of interstitial syndrome, illustrating how AI can enhance ultrasound-based diagnosis across a range of respiratory conditions. These advancements in artifact detection are highly relevant to pneumothorax as well—for instance, confirming the absence of B-lines over an area of interest is part of the ultrasound criteria for PTX (since B-lines are abolished when the pleural layers separate). AI’s ability to reliably recognize such signs can thus reinforce pneumothorax decision-making. By improving the objectivity, consistency, and speed of ultrasound interpretation, AI tools have the potential to transform lung ultrasound into an even more powerful diagnostic modality. Clinicians ranging from emergency physicians and intensivists to aeromedical crews and telemedicine providers could someday rely on AI-augmented ultrasound devices to rapidly rule in or rule out pneumothorax at the point of care, even without extensive sonography training.

Overall, the integration of artificial intelligence with lung ultrasound represents a promising convergence of technologies for improving pneumothorax diagnosis. Ultrasound-based AI models have progressed from initial feasibility studies in animals to sophisticated algorithms approaching expert-level performance in clinical settings. The literature spans a broad spectrum—including algorithmic analyses of lung sliding, deep learning classifiers trained on simulated and real ultrasound data, and prototype systems validated on patients—collectively demonstrating the viability of AI-assisted pneumothorax detection on ultrasound. At the same time, these studies highlight ongoing challenges such as limited public datasets, inter-patient variability, and the need for prospective validation. Further research is warranted to refine the robustness of AI models (e.g., making them more explainable and resistant to image noise), and to evaluate their impact on clinical decision-making and patient outcomes. Ultimately, by reducing operator dependence and standardizing interpretation, reliable AI could enable lung ultrasound to be used more widely and effectively for pneumothorax screening—from busy emergency departments to remote or austere environments where expert sonographers are unavailable.

## 2. Material and Methods

### 2.1. Study Design and Participants

This single-center observational study was conducted between June 2025 and August 2025 after obtaining ethics committee approval (Approval No. 2024-KAEK-64). Written informed consent was obtained from all participants, and the study was carried out in accordance with the Declaration of Helsinki. While lung ultrasound (LUS) data were acquired in real time during the study period, the subsequent evaluation was conducted retrospectively. Inclusion criteria were as follows: adult patients (age ≥ 18 years) who had either a pneumothorax confirmed by chest computed tomography (CT) or no evidence of pneumothorax on CT (healthy controls), with corresponding ultrasound images available for analysis. All included subjects had a diagnostic chest CT performed within 24 h prior to the ultrasound examination to serve as the reference standard for pneumothorax. Exclusion criteria included any cases lacking recorded ultrasound images or with non-diagnostic image quality, as well as patients who had received a pleural intervention (e.g., chest tube placement or needle decompression) before the ultrasound exam (to avoid confounding the ultrasound findings).

A total of 46 participants were included, comprising 23 patients with pneumothorax and 23 healthy controls. The median age was 59 years (Q1–Q3: 41–65.5) in the pneumothorax group and 55 years (Q1–Q3: 41–65.5) in the healthy group, with no significant difference (*p* = 0.964). The proportion of males was identical in both groups (78.3%, *p* = 1.000). Median BMI was 23.0 (Q1–Q3: 20.5–25.5) in the pneumothorax group and 22.0 (Q1–Q3: 21.0–27.0) in the healthy group (*p* = 0.658). The distribution of right-sided involvement was similar between groups (52.2% vs. 56.5%, *p* = 0.767). Smoking prevalence was 65.2% in the pneumothorax group and 69.6% in the healthy group (*p* = 0.753). The presence of chronic obstructive pulmonary disease (COPD) was observed in 34.8% of both groups (*p* = 1.000). No statistically significant differences were found for any demographic or clinical variable between the groups (Table 1). In the pneumothorax group (*n* = 23), the most common etiology was trauma-related pneumothorax (39.1%), followed by secondary spontaneous pneumothorax (SSP) (34.8%) and primary spontaneous pneumothorax (PSP) (26.1%). For image analysis, 60 ultrasound images (combined B-mode and M-mode) were obtained from each participant, resulting in a total of 2760 images included in the study dataset.

### 2.2. Lung Ultrasound Acquisition Protocol

All LUS examinations were performed at the bedside by a single experienced thoracic surgery specialist, using a wireless handheld ultrasound device (Clarius Mobile Health Corp., Vancouver, BC, Canada) set to the manufacturer’s “Lung” preset mode. To standardize image acquisition, the ultrasounds were focused on the lateral chest wall in the “triangle of safety” region—the area commonly used for chest drain insertion (bounded by the lateral border of pectoralis major, the lateral border of latissimus dorsi, and a line at roughly the level of the nipple or 4th–5th intercostal space). Scanning in this region ensured consistent views of the pleural interface for all subjects. In patients with pneumothorax, this approach also facilitated procedural guidance: the ultrasound probe was used to mark an optimal site, and subsequently a pleural decompression (needle thoracostomy or chest tube) was performed at the same location as needed. The success of air aspiration at the ultrasound-marked site provided additional confirmation that a pneumothorax was indeed present in that area (corroborating the CT findings). In the control subjects (no PTX on CT), an identical scanning technique and location were employed for consistency, although no intervention was performed after imaging. For each exam, ultrasound clips and images were recorded and saved in the system for later analysis. During LUS, typical sonographic signs were noted (e.g., lung sliding or its absence, B-lines, etc.), but final determination of pneumothorax for study purposes was based on the reference standard CT results rather than real-time ultrasound interpretation alone.

### 2.3. Reference Standard and Data Collection

**Figure 1:**
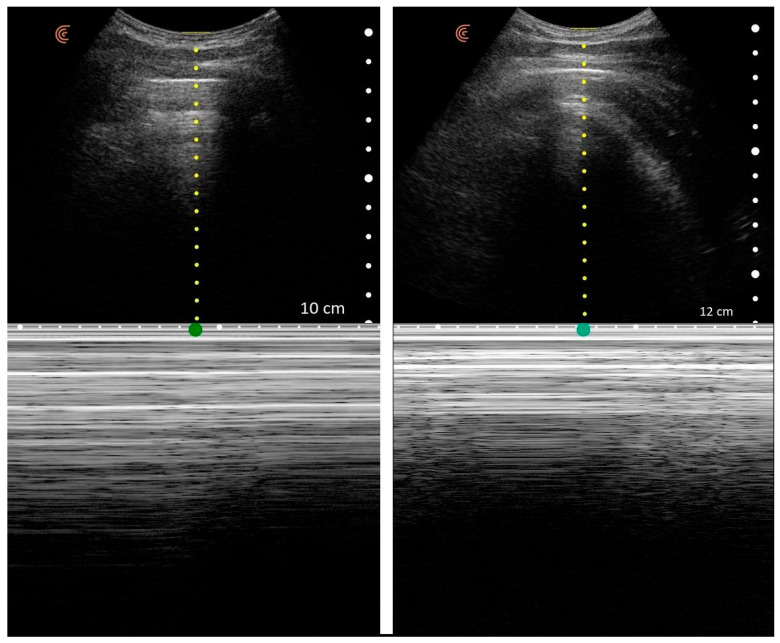
Representative lung ultrasound frames extracted from the dataset. Two examples correspond to the cropped lower region (M-mode, motion mode) and two to the cropped upper region (B-mode, brightness mode), obtained after image pre-processing for model training.

Chest CT was considered the gold standard for diagnosing pneumothorax in this study. All CT scans were interpreted by radiologists as part of routine clinical care, and each case was classified as either “Pneumothorax” or “No Pneumothorax.” These CT findings were used to label the corresponding lung ultrasound (LUS) data, thereby assigning each examination a ground-truth outcome. Notably, CT and ultrasound were performed within a 24 h window, with no therapeutic interventions performed in between, ensuring that the lung condition at the time of LUS corresponded to the CT diagnosis. The first stage of the study involved data collection and labeling in collaboration with Kastamonu University Training and Research Hospital, where a standardized lung ultrasound acquisition protocol was implemented. Each patient record consisted of a 10 s ultrasound video, from which 60 frames were extracted, resulting in a total of 2760 images across all subjects. For subsequent machine learning analysis, a key challenge was that the background regions of the images were disproportionately large compared to the areas of diagnostic interest, which could negatively affect model performance. To address this challenge, the original images were pre-processed to isolate upper and lower regions of interest. Separate datasets were generated for each region, with the number of images remaining identical to the original dataset. Throughout this paper, the cropped bottom region is denoted as M (M-mode, motion mode) and the cropped top region as B (B-mode, brightness mode). Figure 1 illustrates four representative examples, with two images from the B region and two from the M region.

### 2.4. Visual Characteristics of Pneumothorax Images

Lung ultrasound images obtained from pneumothorax patients differ markedly from those of healthy lungs. In normal lungs, B-mode imaging demonstrates the pleural line with dynamic artifacts such as lung sliding and vertical reverberations known as B-lines, while M-mode reveals the characteristic “seashore sign,” reflecting normal pleural motion. In contrast, pneumothorax images typically lack lung sliding and B-lines; instead, the pleural line appears static, and M-mode displays the “barcode sign,” which is diagnostic of absent pleural movement. These visual distinctions form the basis of both clinical interpretation and AI-assisted classification. Representative examples of pneumothorax cases in B-mode and M-mode are illustrated in Figure 1.

### 2.5. Statistical Analysis

Statistical analyses were performed using IBM SPSS Statistics, version 23 (IBM Corp., Armonk, NY, USA). Continuous variables (age and BMI) were expressed as median and interquartile range (Q1–Q3) and compared between groups using the Mann–Whitney U test due to non-normal distribution. Categorical variables were presented as counts and percentages, and between-group comparisons were performed using Pearson’s chi-square test without continuity correction. A two-tailed *p*-value < 0.05 was considered statistically significant.

### 2.6. Transformers

Transformers employ self-attention mechanisms to emphasize the most relevant elements of input sequences, making them highly efficient across a wide range of tasks.

The self-attention mechanism [26] which differs from the classical attention mechanism, is the core of the transformer. This mechanism dynamically allocates attention weights by calculating the correlation between each element in the input and other elements [27,28]. As a result, the self-attention mechanism effectively processes global information and identifies the most important elements of input sequences [27]. In this framework, this architecture generates context-integrated representations [28] that improve the model’s capacity to extract global features.

Transformer-based models can also be enhanced through knowledge distillation, whereby information from a larger pretrained teacher model is transferred to a more compact student model, enabling faster training without compromising accuracy. Vision Transformers, in particular, have gained prominence due to their strong capability in processing visual inputs [29]. Recent studies have highlighted their successful application in medical imaging tasks, including radiology, pathology, and lung ultrasound, where they demonstrate superior performance compared with conventional convolutional neural networks [29,30]. Building on this evidence, we applied transformer-based architectures to lung ultrasound images in order to evaluate their diagnostic utility for pneumothorax detection.

#### 2.6.1. Vision Transformer

**Figure 2:**
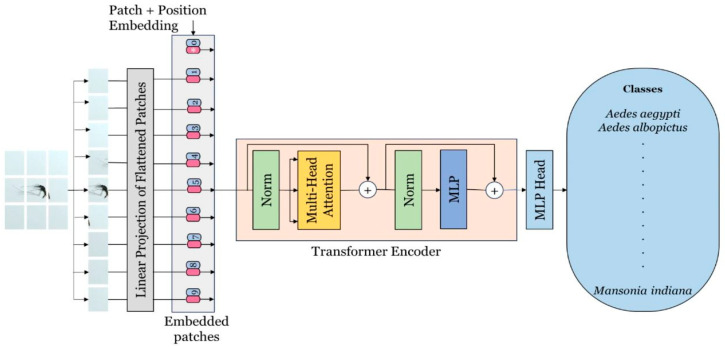
Overview of the Vision Transformer (ViT) architecture, consisting of patch embedding, transformer encoder, and a multi-layer perceptron (MLP) head for binary classification. Adapted from Kittichai et al., 2024 [33], licensed under CC BY-NC-ND 4.0.

The Vision Transformer (ViT) is a modern deep learning framework tailored for visual recognition applications. It marks a significant innovation by adapting transformer architectures—originally created for natural language processing—to the domain of computer vision [31]. By applying the transformer mechanism to images, ViT is able to capture both local and global contextual relationships within visual data [32]. The architecture is organized into three primary components: Patch Embedding, the Transformer Encoder, and the Multi-Layer Perceptron (MLP) Head. In the Patch Embedding stage, an image is partitioned into fixed-size patches that are linearly projected and enriched with positional encodings before being passed into the Transformer Encoder. This encoder, which plays a central role in feature extraction, replaces the need for a convolutional backbone and is composed of multiple layers containing multi-head self-attention and feed-forward submodules, each followed by normalization. The MLP Head forms the final stage and includes two fully connected hidden layers with 2048 and 1024 units, respectively, which contribute to the classification process. The output layer then produces two logits corresponding to the binary classes of the pneumothorax dataset. A schematic representation of the ViT architecture is depicted in Figure 2 [33].

#### 2.6.2. DINOv2

**Figure 3:**
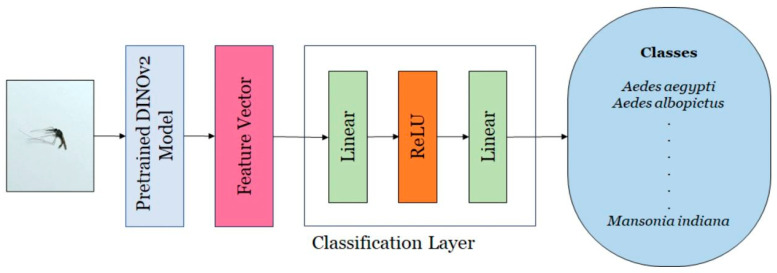
Overview of the DINOv2 architecture illustrating its backbone, feature extraction process, and classification head. Adapted from Kittichai et al., 2024 [33], licensed under CC BY-NC-ND 4.0.

DINOv2 is a self-supervised variant of the ViT that produces spatially aware features suitable for dense prediction tasks. For each image, it generates a global token together with a grid of local patch tokens. The PolaCount module preserves patch-level placements, as these localization cues are essential for accurate object counting [34]. This architecture integrates self-distillation into its training strategy and functions as a backbone network for feature extraction. This backbone employs a transformer-based architecture designed to derive high-dimensional feature representations from input images. The extracted features have been shown to outperform task-specific models and state-of-the-art baselines in various downstream applications. A classification head is subsequently attached to the backbone, using the feature vector to perform image-level classification. The classifier consists of two fully connected layers: the first projects the feature space to a 256-dimensional embedding, followed by a second layer that maps it to the number of target classes. ReLU activation is applied after the first linear transformation. Figure 3 provides a schematic overview of the DINOv2 architecture, illustrating its core components and interactions [33].

### 2.7. Video Vision Transformer (ViViT)

**Figure 4:**
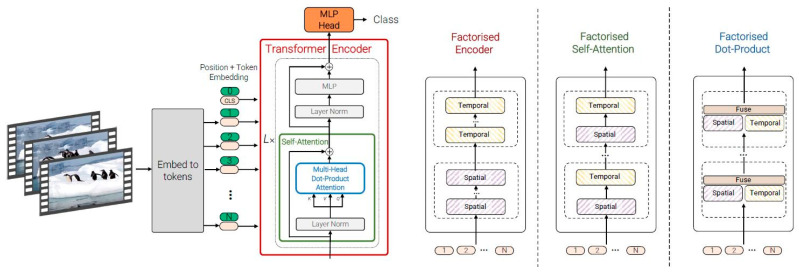
Schematic representation of the ViViT architecture for video-based pneumothorax detection. The model divides each ultrasound frame into patch tokens and processes them through multi-head self-attention layers to capture spatial–temporal dependencies across ultrasound sequences. Adapted from Arnab et al., 2021 [35], licensed under CC BY 4.0.

ViViT is a video recognition model based on a transformer structure. It divides each frame into patch units and encodes them through a transformer encoder to extract features from the entire video. Accordingly, this modeling learns the relationships between patches through a multi-head attention mechanism and outputs the final recognition result through MLP layers [35]. The ViViT architecture uses a multi-head self-attention mechanism to capture multiple relationships between tubes in different subspaces. The attention mechanism is applied independently for each head, and the results are combined. Moreover, the capacity to preserve volumetric context facilitates more precise and consistent predictions across diverse patient data [36]. Figure 4 presents a schematic overview of the ViViT architecture.

### 2.8. Evaluation Metrics

In a binary classification task, model performance can be assessed using a 2 × 2 confusion matrix, which includes true positives (TP), true negatives (TN), false positives (FP), and false negatives (FN). In this matrix, the rows represent the actual classes, while the columns indicate the predicted classes. Several standard metrics are derived from these values. Accuracy reflects the proportion of correctly classified cases (both positive and negative) relative to the total number of samples. Recall (sensitivity) measures the proportion of true positive cases correctly identified out of all actual positives. Precision denotes the proportion of true positive predictions among all cases predicted as positive. F1-score represents the harmonic mean of precision and recall, providing a balanced measure of a model’s reliability in both detecting positive cases and minimizing false alarms.

(1) $$Accuracy=\frac{TP+TN}{TP+FN+TN+FP}$$

(2) $$Recall=\frac{TP}{TP+FN}$$

(3) $$Precision=\frac{TP}{TP+FP}$$

(4) $$F1-score=\frac{2\times Precision\times Recall}{Precision+Recall}$$

### 2.9. Experimental Setup and Training Details

**Figure 5:**
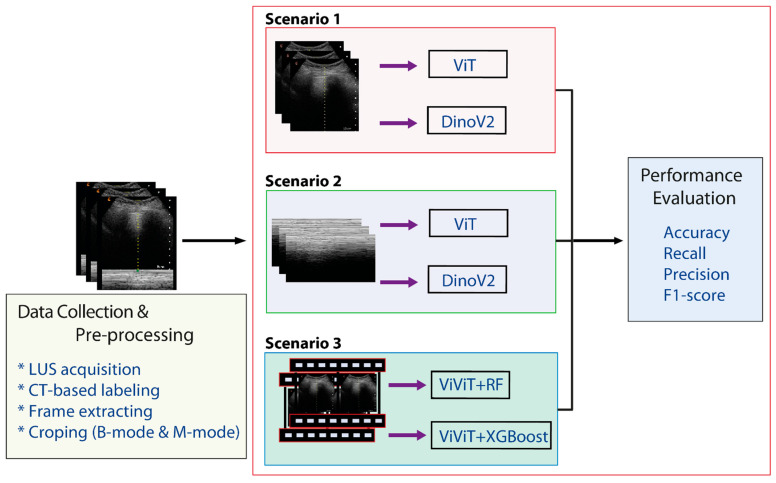
Experimental workflow for Pneumothorax detection using AI-assisted lung ultrasound.

All deep learning experiments were conducted in a CUDA-enabled Google Colaboratory environment using the Keras framework, which provided efficient processing and faster training. Given the limited number of class-level samples in the dataset, we employed pretrained transformer-based architectures-ViT and DINOv2—rather than constructing models from scratch. Also, ViViT architecture was used for extraction of feature maps from video sequences. ViViT experiment was conducted using the ‘Hugging Face’ library. This library is one of the most widely used tools for the transformer library, natural language processing, computer vision, and deep learning models. No architectural modifications were made to the original models. Figure 5 summarizes the main stages of the experimental workflow: (a) data collection and pre-processing, (b) model training, and (c) performance evaluation.

To prevent potential overfitting due to repeated frames in the 10 s video sequences, subsets were generated by selecting one out of every five frames (e.g., 1st, 6th, 11th, etc.) from each patient. Although the initial dataset contained 2760 frames (60 frames per patient × 46 patients), this down-sampling strategy reduced the number to 552 representative images. Three scenarios were implemented to validate the performances of deep learning models. In Scenario 1, this dataset was randomly divided into training, validation, and test sets, resulting in 342 images (62%) for training, 99 images (17.9%) for validation, and 111 images (20.1%) for testing. While this random split achieved high accuracy, it carried the risk of data leakage, as frames from the same patient could appear across different subsets. In Scenario 2, patient-level separation was strictly enforced. A total of 26 patients (312 images, 56.6%) were allocated to training, 10 patients (120 images, 21.7%) to validation, and another 10 patients (120 images, 21.7%) to testing. This ensured that no individual contributed images to more than one subset, thereby improving the robustness of the evaluation and eliminating overlap across sets. In Scenario 3, the performances of the RF and XGBoost classifiers were evaluated using feature maps extracted from video sequences with the ViViT architecture. In this case, 80% of the 46 patient videos were designated for training, with the remainder for testing. As a result, 36 patients were allocated for training and 10 for testing. Table 2 summarizes the information about the train, validation, and testing sets for both scenarios.

All images were resized according to the input requirements of each architecture in two scenarios. Also, the normalization, resizing, random flip (“horizontal”), random rotation (factor = 0.02), random zoom (height factor = 0.2, width factor = 0.2) data augmentation techniques were applied on the training sets for the training of ViT and DinoV2 networks. Both models were trained for 30 epochs with a batch size of 256, using the Adam optimizer with an initial learning rate of 1 × 10^−3^. Hyperparameters and important techniques for all models are summarized in Table 3 and Table 4. The performances of the models were evaluated using accuracy, sensitivity, precision, F1-score, and AUC.

## 3. Results

**Figure 6:**
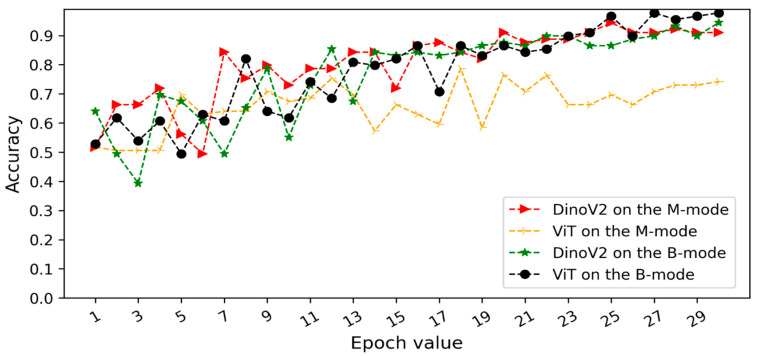
Validation accuracy curves of transformer models (DINOv2 and ViT) for pneumothorax detection under Scenario 1 (random frame split). The “M-mode region” corresponds to the motion-mode area below the pleural line, while the “B-mode region” represents the pleural line and overlying structures in brightness-mode imaging.

**Figure 7:**
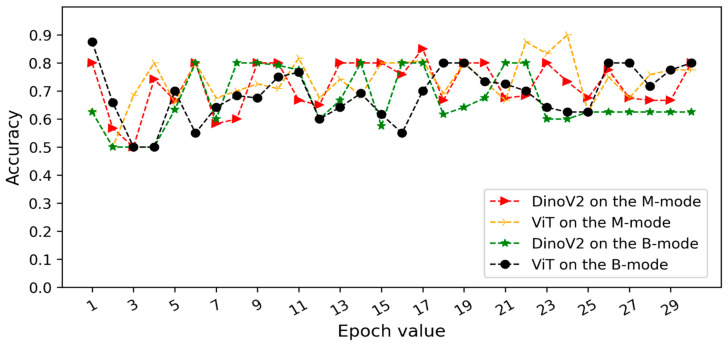
Validation accuracy curves of transformer models (DINOv2 and ViT) for pneumothorax detection under Scenario 2 (patient-level split). The “M-mode region” corresponds to the motion-mode area below the pleural line, while the “B-mode region” represents the pleural line and overlying structures in brightness-mode imaging.

This section presents a comprehensive summary of model performance during training, validation, and test set evaluation for automatic pneumothorax detection. The experiments were conducted under three scenarios. In the first two scenarios, M-mode images derived from the region with lower motion and B-mode images focused on the pleural line region were used, and deep learning models were compared to assess their ability to distinguish pneumothorax from healthy lungs. Figure 6 and Figure 7 illustrate the validation accuracy curves over training epochs for each model. Particularly, Figure 6 corresponds to Scenario 1 and Figure 7 to Scenario 2. In both datasets, the models achieved consistently high accuracy, with B-mode (pleural line) inputs generally yielding overall superior performance.

**Figure 8:**
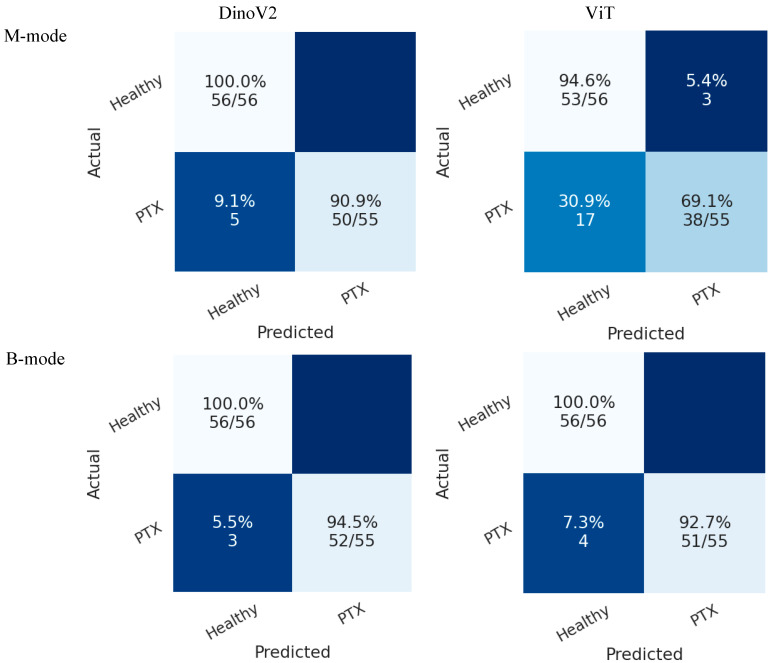
Confusion matrices of transformer models (DINOv2 and ViT) for pneumothorax detection in Scenario 1. Each cell shows classification accuracy as a percentage, with the corresponding absolute number of correctly classified cases over total cases in parentheses. Top-left = TN, top-right = FP, bottom-left = FN, bottom-right = TP.

Figure 8 presents the confusion matrices of deep learning models on the test sets in Scenario 1. In the B-mode dataset, both ViT and DINOv2 demonstrated superior performance compared with the M-mode dataset. In this scenario, the DINOv2 model misclassified only 3 pneumothorax images in the B-mode region, while the ViT model misclassified 4 images. Both models achieved perfect classification of healthy cases.

**Figure 9:**
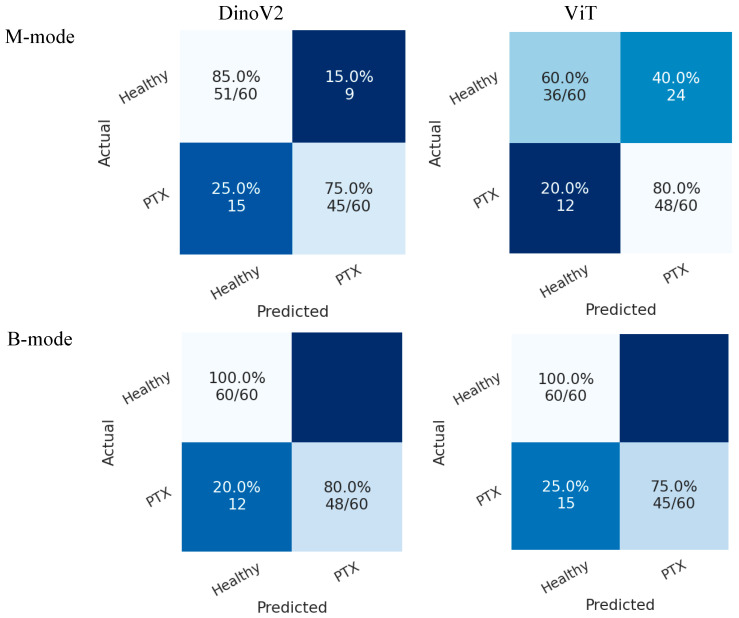
Confusion matrices of transformer models (DINOv2 and ViT) for pneumothorax detection in Scenario 2. Each cell shows classification accuracy as a percentage, with the corresponding absolute number of correctly classified cases over total cases in parentheses. Top-left = TN, top-right = FP, bottom-left = FN, bottom-right = TP.

In Scenario 2, both models also achieved higher accuracy in the B-mode region compared with the M-mode region. As shown in Figure 9, the DINOv2 model misclassified 12 pneumothorax images, while the ViT model misclassified 15 images. Both models achieved perfect classification of healthy cases.

**Figure 10:**
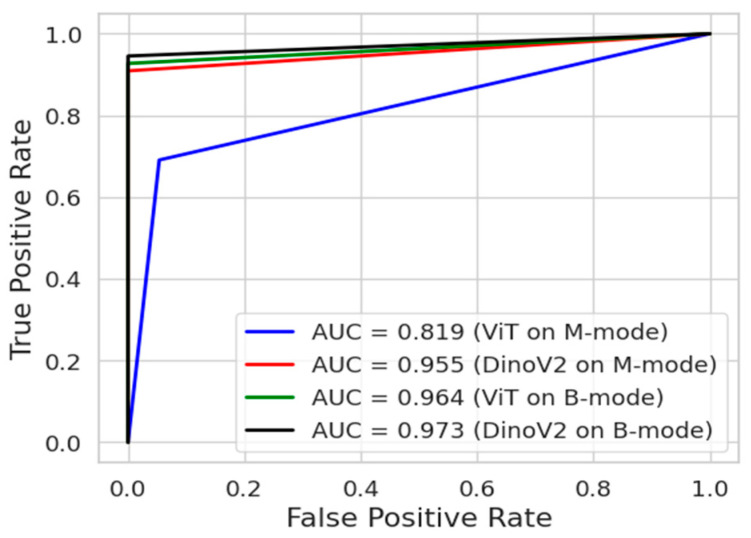
ROC analysis of transformer models (DINOv2 and ViT) for Pneumothorax detection in the first two scenarios.

Figure 10 presents the ROC curves of the deep learning models. The ROC curve is an important tool frequently used in medical data analysis fields. This curve visualizes the balance between sensitivity and specificity offered by a classifier model. The blue and red colours indicate the ROC curves of models trained on M-mode images. On the other hand, the green and black colours indicate the ROC curves of models trained on B-mode images. As can be seen, the DinoV2 and ViT models achieved an AUC of 0.973 and 0.964, respectively, on B-mode images.

**Figure 11:**
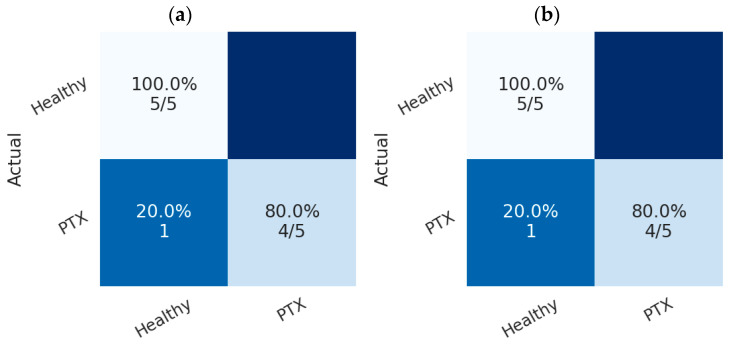
Confusion matrices of the classifiers on the feature maps extracted from video sequences obtained using ViViT feature extractor; (**a**) RF, (**b**) XGBoost.

**Figure 12:**
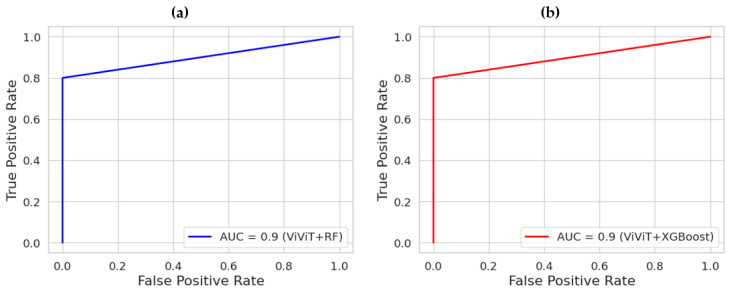
ROC results of the classifiers on the feature maps using ViViT feature extractor; (**a**) RF, (**b**) XGBoost.

Scenario 3 presents ViViT experiments for detecting Pneumothorax using ultrasound data. The ViViT model processes the total ultrasonic volume by analyzing the temporal frames in a sequential video sequence. This allows the model to automatically learn critical spatiotemporal patterns representing relationships in ultrasound data. The ViViT architecture minimizes model fragmentation and the risk of error propagation by directly processing temporal frames from the B-mode ultrasound video sequences. The performances of RF and XGBoost classifiers were investigated on feature maps extracted from ultrasound video sequences using the ViViT transformer architecture. Accordingly, 80% of the dataset, which includes feature maps obtained from ultrasound video data of 46 patients, was allocated for training these classifiers, and 20% for validating their performance. Both classifiers yielded very successful results. Figure 11 presents the confusion matrices of the classifier models on the ViViT features for testing set in Scenario 3. Accordingly, the RF and XGBoost classifiers achieved an overall success rate of 90% in detecting Pneumothorax. Also, Figure 12 presents the ROC curves of the classifiers on the feature maps obtained using the ViViT feature extractor. Both classifiers presented an AUC value of 0.9.

Table 5 presents the performance of the deep learning models in three scenarios. The results from both the M-mode and B-mode regions indicate that both models performed better in the B-mode region compared with the M-mode region. Bold values indicate the highest metric values. In Scenario 1, the ViT model achieved the highest accuracy in the B-mode region, with 99.1%, while the DINOv2 model also demonstrated acceptable performance with 97.3% accuracy in the same region. In Scenario 2, the DINOv2 model achieved the best performance in the B-mode region with 90% accuracy, corresponding to 90% accuracy, 80% recall, 100% precision, and 88.89% F1-score. In addition, RF and XGBoost classifiers presented 90% accuracy, 80% recall, 100% precision and 88.89% F1-score on ViViT feature maps in Scenario 3.

Overall evaluation is as follows: In scenario 1, images of the same patient were included in both the training and test sets. With this in mind, we also conducted experimental studies using the second scenario to prevent data leakage. Thus, the trained model’s performance on previously unseen images was examined. The ViT and DinoV2 models trained on B-mode ultrasound images of different patients achieved accuracy scores of 87.5% and 90.0%, respectively. The curves for validation datasets (Figure 6 and Figure 7) show stable convergence, confirming that there is no overfitting. The results indicate that both models produced consistent results without being negatively affected by the small number of patients. 90% accuracy on the test set demonstrates that the model has the capacity to generalize to a dataset it had not seen during training. As a result, the DinoV2 architecture in Scenario 2, where the same patients are not included in both the training and test sets, demonstrates high resistance to memorization. Furthermore, the robustness of the model is further strengthened in Scenario 2 compared to Scenario 1. In addition, the ViViT model presented high accuracy on the patients’ video sequences and was also quite successful in Scenario 3.

## 4. Discussion

### 4.1. Comparison with Existing Literature

The findings of our study are largely consistent with recent literature on AI-assisted lung ultrasound (AI-LUS) for pneumothorax detection. Multiple studies in recent years have demonstrated that deep learning models can achieve near-expert performance when applied to lung ultrasound for pneumothorax diagnosis. For instance, VanBerlo et al. [17] trained a deep learning model on large-scale LUS datasets from two academic centers and reported 93.5% sensitivity and 87.3% specificity, with similar performance on an external dataset, indicating good generalizability. Likewise, Qiang et al. [18] introduced a spatiotemporal deep learning model capable of analyzing full LUS video clips, achieving 96.9% accuracy, 99.2% sensitivity, and 89.2% specificity, representing one of the highest-performing systems to date. These results corroborate our finding that transformer-based architectures, particularly DINOv2, can deliver high diagnostic accuracy, with patient-level splits in our study yielding AUC values around 0.97, comparable to the best values reported in the literature.

Earlier approaches showed more modest performance. Rule-based algorithms initially reported sensitivities of around 79%, whereas deep learning models, when carefully trained to avoid data leakage, achieved marked improvements. Jascur et al. [11] applied a ResNet-based model to post-thoracic surgery patients, reporting 82% sensitivity and 92% specificity. Similarly, Montgomery et al. [14] demonstrated that AI-assisted ultrasound could detect pneumothorax with 86% sensitivity and 75% specificity. Our study further confirms that B-mode images are more informative than M-mode, consistent with reports emphasizing the limitations of frame-based analysis when temporal pleural motion is underutilized. Importantly, by applying a strict patient-level split, our study mitigated data leakage—a methodological issue that inflated performance in some earlier studies.

Prospective evaluations also highlight the potential of AI in real-world clinical practice. Clausdorff Fiedler et al. [24] reported that an AI tool achieved 90–100% sensitivity in real-time lung sliding detection in trauma patients, though specificity was moderate in low-quality scans. This mirrors the sensitivity-specificity tradeoff observed in our models, where DINOv2 maximized specificity (100%) at the expense of slightly lower sensitivity in the patient-level scenario. Yang et al. [25] found that AI-assisted LUS demonstrated 79.4% sensitivity and 85.4% specificity, comparable to chest radiography in the same cohort. Taken together, these reports align with our results, reinforcing the conclusion that transformer-based models can achieve diagnostic accuracy close to that of human experts while avoiding methodological pitfalls.

### 4.2. Clinical Implications

Our findings underscore the value of AI-assisted LUS in scenarios where access to radiologists or expert sonographers is limited. In emergencies such as traumatic pneumothorax, prehospital personnel—including paramedics in ambulances or general practitioners in rural clinics—may be the first and only providers available. Traditional lung ultrasound is highly operator-dependent, and inexperience can significantly limit diagnostic accuracy. AI integration can mitigate this limitation by functioning as a standardized observer, automatically detecting sonographic signs of pneumothorax.

Evidence from diverse ultrasound applications underscores this potential. A multicenter investigation showed that AI-assisted lung ultrasound enhanced diagnostic accuracy and improved agreement among less experienced clinicians. Likewise, Sultan et al. [37] reported that AI-driven teleguided point-of-care ultrasound enabled individuals without prior ultrasound training to effectively perform scans for COVID-19 pneumonia. Collectively, these findings highlight how AI can broaden the clinical utility of LUS, making it accessible to non-specialist providers.

AI-assisted LUS also holds promise beyond hospitals. In prehospital settings such as ambulances, battlefield medicine, or maritime environments, handheld ultrasound devices equipped with AI algorithms could allow rapid, accurate diagnosis of pneumothorax even in the absence of a physician. Case reports have already described successful tele-ultrasound-guided pneumothorax detection by paramedics. With AI integration, such workflows could be further streamlined, reducing dependence on remote specialists. Thus, the combination of portable devices and embedded AI has the potential to democratize access to high-quality imaging, bridging the diagnostic gap between tertiary centers and austere environments.

Beyond pneumothorax detection, the proposed AI-based framework could be extended to identify other thoracic abnormalities such as pleural effusion, lung consolidation, or pulmonary edema, supporting broader applications in critical care ultrasound.

### 4.3. Strengths and Limitations

This study has several strengths. First, pneumothorax diagnoses were confirmed with CT, ensuring a robust reference standard. Second, the use of a strict patient-level split eliminated the risk of data leakage, a known source of bias in prior studies. Third, the evaluation of transformer-based architectures, which are less commonly applied to lung ultrasound, provides novel insights into their diagnostic utility.

Nonetheless, important limitations should be noted. The single-center, retrospective design and modest sample size (*n* = 46) may restrict generalizability. All images were acquired by a single operator using one handheld device, reducing variability but excluding inter-operator and inter-device effects. Frame-based analysis limited the exploitation of temporal pleural motion, which could be better captured by video-based models such as temporal shift networks. Although CT and LUS were performed within 24 h, minor clinical changes during this interval cannot be excluded. A practical limitation was the absence of a high-frequency linear probe during data collection. The handheld curvilinear transducer—chosen for portability, consistent bedside access in the ED/ICU, and for marking the “triangle of safety” in procedures—was used to standardize acquisition and ensure adequate penetration across body habitus. Notably, for pneumothorax detection the core signs (absent lung sliding on B-mode and the “barcode” pattern on M-mode) can be reliably assessed with either linear or curvilinear probes. Still, prior reports suggest that while overall differences between probe types are minimal, small pneumothoraces may be more easily detected with high-frequency linear transducers [38]. Finally, models were trained with fixed hyperparameters and single-vendor images, potentially limiting out-of-distribution performance.

An additional limitation is the absence of explainability methods such as saliency mapping, Grad-CAM, or attention heatmaps, which are increasingly recommended to support clinical trust in AI-assisted diagnostic systems. Future work should incorporate visualization of model decision pathways at the pleural line to provide transparency for clinicians and to facilitate adoption in practice.

Future research should therefore pursue multicenter, prospective validation across diverse patient populations, devices, and probe types. Video-based models, calibration methods, and explainability features targeted at the pleural line may further enhance clinical trust and usability. Ultimately, pilot workflow studies integrating AI-assisted LUS into trauma protocols such as E-FAST will be essential to establish real-world feasibility and impact.

## 5. Conclusions

This study demonstrates that state-of-the-art deep learning models can significantly enhance the diagnostic accuracy of lung ultrasound for pneumothorax detection. By systematically comparing transformer-based and convolutional architectures, we found that transformer models, particularly DINOv2, achieved the highest performance across multiple evaluation metrics. These findings suggest that AI-assisted analysis has the potential to reduce operator dependency, improve diagnostic consistency, and support timely decision-making in emergency and critical care settings. The results provide a foundation for the integration of artificial intelligence into point-of-care ultrasound workflows, highlighting its promise as a valuable adjunct to clinical expertise in pneumothorax diagnosis.

To our best knowledge, this is the first study that employs transformer architectures for ultrasound imaging and ultrasound video sequences and presents consistent and competitive results. The proposed method’s high success rate on both the training and test datasets gives significant information to healthcare providers. The findings are expected to lead to the development of a secure and clinically relevant system. The ultimate goal is to create a user-friendly application that runs a successful deep learning model and gives expert decision support via a user interface, potentially lowering expert workload and contributing to the balance of early diagnosis and treatment. In future studies, this approach may also be adapted for other lung ultrasound pathologies to further enhance point-of-care diagnostic workflows. Furthermore, in the near future, we plan to improve the suggested model by performing k-fold cross-validation and external validation on the datasets from various sources.

## Figures and Tables

**Table 1:** Demographic and clinical characteristics of the study groups. BMI—Body Mass Index; Q1—first quartile; Q3—third quartile.

	Pneumothorax (*n* = 23)	Healthy (*n* = 23)	*p*-Value
Age, median (Q1–Q3)	59 (41–65.5)	55 (41–65.5)	0.964
Male, *n* (%)	18 (78.3)	18 (78.3)	1
BMI, median (Q1–Q3)	23 (20.5–25.5)	22 (21–27)	0.658
Right Sided, *n* (%)	12 (52.2)	13 (56.5)	0.767
Smoking, *n* (%)	15 (65.2)	16 (69.6)	0.753
COPD, *n* (%)	8 (34.8)	8 (34.8)	1

**Table 2:** Dataset information for Scenario 1 (random split), Scenario 2 (patient-level split) and Scenario 3 (patient-level video sequences in B-mode).

Dataset	Scenario 1	Scenario 2	Scenario 3
Training	342 (62%)	312 (56.6%)	36 patients (78.2%)
Validation	99 (17.9%)	120 (21.7%)	-
Test	111 (20.1%)	120 (21.7%)	10 patients (21.8%)
Total	552 (100%)	552 (100%)	46 patients (100%)

**Table 3:** Hyperparameters used for training the ViT and DinoV2 transformer architectures.

Learning rate	0.001
Optimizer	Adam
Loss function	Sparse Categorical Cross Entropy
Weight decay	0.0001
Batch size	256
Number of epochs	30
Image size	72
Patch size	6
Checkpoint	monitor = “validation accuracy”
Early Stopping	patience = 15, monitor = ‘validation loss’

**Table 4:** Hyperparameters used for ViViT feature extractor.

Image size	224
Number of frames	32
Hidden size	768
Number of hidden layers	12
Number of attention heads	12
Hidden activation function	‘gelu fast’
Tubelet size	[2, 16, 16]
Initializer range	0.02
Layer norm eps	1 × 10^−6^

**Table 5:** Comparative performance of transformer models (DINOv2 and ViT) in pneumothorax classification across Scenario 1 and Scenario 2 using M-mode and B-mode image regions. Acc—accuracy; Rec—recall (sensitivity); Pre—precision; F1-score—harmonic mean of precision and recall. Bold values indicate the highest metric for each scenario.

Scenario	Mode	Models	Acc	Rec	Pre	F1-Score
1	M-mode	DinoV2	95.5	90.91	100.0	95.24
		ViT	81.98	69.09	92.68	79.17
	B-mode	DinoV2	97.3	94.55	100.0	97.2
		ViT	99.1	98.21	100.0	99.1
2	M-mode	DinoV2	80.0	75.0	83.33	78.95
		ViT	70.0	80.0	66.67	72.73
	B-mode	DinoV2	90.0	80.0	100.0	88.89
		ViT	87.5	75.0	100.0	85.71
3	Video sequences in B-mode	ViViT + RF	90.0	80.0	100.0	88.89
		ViViT + XGBoost	90.0	80.0	100.0	88.89

## Data Availability

The data presented in this study are available on reasonable request from the corresponding author. The data are not publicly available because of institutional policy and ethical considerations.
